# A variant upstream of *HLA-DRB1* and multiple variants in *MICA* influence susceptibility to cervical cancer in a Swedish population

**DOI:** 10.1002/cam4.183

**Published:** 2014-01-07

**Authors:** Dan Chen, Joanna Hammer, David Lindquist, Annika Idahl, Ulf Gyllensten

**Affiliations:** 1 Department of Immunology, Genetics and Pathology, Rudbeck Laboratory, Science for Life Laboratory Uppsala, Uppsala UniversitySE-751 85, Uppsala, Sweden; 2 Department of Radiation Sciences, Umeå UniversitySE-901 87, Umeå, Sweden; 3 Department of Clinical Sciences, Obstetrics and Gynecology, Umeå UniversitySE-901 87, Umeå, Sweden

**Keywords:** Cervical cancer, *cis*-eQTL, frameshift mutation, *HLA-DRB1*, *MICA*

## Abstract

In a genome-wide association study, we have previously identified and performed the initial replication of three novel susceptibility loci for cervical cancer: rs9272143 upstream of *HLA-DRB1*, rs2516448 adjacent to MHC class I polypeptide-related sequence A gene *(MICA),* and rs3117027 at *HLA-DPB2*. The risk allele T of rs2516448 is in perfect linkage disequilibrium with a frameshift mutation (A5.1) in *MICA* exon 5*,* which results in a truncated protein. To validate these associations in an independent study and extend our prior work to *MICA* exon 5, we genotyped the single-nucleotide polymorphisms at rs9272143, rs2516448, rs3117027 and the *MICA* exon 5 microsatellite in a nested case–control study of 961 cervical cancer patients (827 carcinoma in situ and 134 invasive carcinoma) and 1725 controls from northern Sweden. The C allele of rs9272143 conferred protection against cervical cancer (odds ratio [OR] = 0.73, 95% confidence interval [CI] = 0.65–0.82; *P* = 1.6 × 10^−7^), which is associated with higher expression level of *HLA-DRB1*, whereas the T allele of rs2516448 increased the susceptibility to cervical cancer (OR = 1.33, 95% CI = 1.19–1.49; *P* = 5.8 × 10^−7^), with the same association shown with *MICA-A5.1*. The direction and the magnitude of these associations were consistent with our previous findings. We also identified protective effects of the *MICA-A4* (OR = 0.80, 95% CI = 0.68–0.94; *P* = 6.7 × 10^−3^) and *MICA-A5* (OR = 0.60, 95% CI = 0.50–0.72; *P* = 3.0 × 10^−8^) alleles. The associations with these variants are unlikely to be driven by the nearby human leukocyte antigen (HLA) alleles. No association was observed between rs3117027 and risk of cervical cancer. Our results support the role of *HLA-DRB1* and *MICA* in the pathogenesis of cervical cancer.

## Introduction

Worldwide, cervical cancer is the third most common cancer and the second most frequent cause of cancer deaths among women, and resulted in an estimated 530,000 new cancer cases and 275,000 deaths in 2008 1. In many low-income countries, cervical cancer is the most common cancer and the leading cause of cancer-related death among women 1. Cervical cancer and its precursor lesions, cervical intraepithelial neoplasia (CIN) are caused by persistent infection with high-risk human papillomavirus (HPV), where CIN III is considered the same as carcinoma in situ (CIS) 2. During their lifetime, many women will become infected with HPV, but only a minority will develop CIN or cervical cancer. Consequently, other factors, for example, host genetic factors, play an important role in both the persistence of infection and progression to cancer 3,4.

We have recently performed the first genome-wide association study (GWAS) of cervical cancer and identified three independent novel loci within the major histocompatibility complex (MHC) region at 6p21.3 that influence susceptibility to cervical cancer in a Swedish population. The first is located between *HLA-DRB1* and *HLA-DQA1* (rs9272143; odds ratio [OR] = 0.67, 95% confidence interval [CI] = 0.62–0.72 for C allele; *P* = 9.3×10^−24^); the second is adjacent to the MHC class I polypeptide-related sequence A gene *(MICA)* (rs2516448; OR = 1.42, 95% CI = 1.31–1.54 for T allele; *P* = 1.6 × 10^−18^); and the third at *HLA-DPB2* (rs3117027; OR = 1.25, 95% CI = 1.15–1.35 for A allele; *P* = 4.9×10^−8^) 5. The associations observed for these three new loci were found to be statistically independent of previously known associations with the human leukocyte antigen (HLA) alleles/haplotypes 5. The transmembrane domain (TMD) of MICA encoded by exon 5 harbors a variable number of GCT repeats, which encode 4, 5, 6, or 9 alanine (Ala) residues (alleles designated A4, A5, A6 or A9, respectively). Additionally, the A5.1 allele (rs67841474) contains an extra guanine (G) insertion after two GCT triplets, which causes a frameshift mutation resulting in a premature stop codon that, in turn, truncates 10 amino acids of the TMD as well as the hydrophobic cytoplasmic tail 6. The risk allele T of rs2516448 was found to be in perfect linkage disequilibrium (LD) (*D' *= 1, *r*^2^ = 1) with A5.1 and cervical neoplasia patients carrying the A5.1 allele have less membrane-bound MICA in their lesions 5.

In our initial GWAS, we were able to replicate the effect of the three susceptibility loci in a second cohort from southern and middle Sweden. However, validation of GWAS findings in multiple cohorts is necessary in order to report genotype–phenotype associations. The new susceptibility loci for cervical cancer require further investigation in a large sample size. It is also important to extend our prior work to *MICA* exon 5 microsatellite polymorphism and evaluate effect modification by age of onset and tumor stage. Therefore, we investigated the association between single-nucleotide polymorphisms (SNPs) of rs9272143, rs2516448, and rs3117027 as well as *MICA* exon 5 microsatellite polymorphism and risk of cervical cancer, in a large nested case–control study of 961 incident cervical cancer patients (827 CIS and 134 invasive carcinoma) and 1725 cancer-free controls from the Västerbotten County in northern Sweden.

## Material and Methods

### Study population

Eligible women for the study were defined as Västerbotten County resident in northern Sweden who had at least one cytologically normal cervical smear and who had no prior operative treatment of the cervix. Linkage between the cytology registry and the Swedish Cancer Registry from 1961 identified 832 patients with CIS and 134 patients with invasive cervical cancer diagnosed after the sampling date of a normal smear. Controls were women in the study base who did not develop cervical cancer before the time point of diagnosis of a corresponding case. For each CIS case, two population-based controls were selected, matched for age of subject (±5 years) when the sample was collected. For each invasive cervical cancer case, one population-based control was selected, matched for age of subject (±5 years) when the sample was collected. A written informed consent was obtained from each participant and this study was approved by the Institutional Review Board (IRB) of the Umeå University. Genomic DNA was extracted from the buffy coat using standard phenol–chloroform extraction protocol. In total, DNA samples from 827 women with CIS and 1591 matched healthy controls, and 134 women with invasive cervical cancer and 134 matched healthy controls qualified for genotyping. The study population was not included in the previous GWAS study.

### Genotyping assay

Single-nucleotide polymorphisms of rs9272143 and rs3117027 were genotyped with template-directed dye-terminator incorporation with fluorescence polarization detection (FP-TDI) (Tecan, Männedorf, Switzerland) and rs2516448 was genotyped using the TaqMan assay (Applied Biosystems, Foster City, CA). The information of the primers and probes is described in Supplementary Table S1. The polymerase chain reaction (PCR) amplification of the *MICA* microsatellite alleles of exon 5 was carried out using a 5′ end fluorescently (6-FAM)-labeled reverse primer and a forward unlabeled primer. The primer sequences for the *MICA* microsatellite were previously reported 7 and are described in Supplementary Table S1. The PCR products were mixed with Hi-Di Formamide and GeneScan 500 ROX size standard and separated on an ABI 3730xl DNA Analyzer (Applied Biosystems). Different alleles were annotated using GeneMapper 4.1 software (Applied Biosystems, Foster City, USA) based on the size of the PCR products. Eight percent of the samples were selected for repeat genotyping as duplicates, yielding a reproducibility of 100%. Genotype success rate was >98.21%.

**Table 1:** Selected demographic characteristics of study subjects

	Cases *N*^2^ (%)	Controls *N*^2^ (%)	*P*^3^
Age (years)^1^			
<36	478 (49.33)	851 (49.74)	0.22
≥36	483 (50.67)	874 (50.26)	
Mean± SD	36.86 ± 9.05	36.43 ± 8.86	
Study design			
Study 1	827^4^ (86.06)	1591 (92.23)	
Study 2	134^5^ (13.94)	134 (7.77)	
Total	961	1725	

SD, Standard error.

1 The median age is 36 years for both cervical cancer patients and control subjects.

2 Number of samples.

3 Difference in age between cervical cancer patients and control subjects was evaluated by using *t*-test. *P* value is two-sided.

4 Number of subjects with carcinoma in situ.

5 Number of subjects with invasive carcinoma.

### Statistical analyses

Differences in age between the cervical cancer cases and controls were evaluated by using t-test. Goodness-of-fit to the Hardy–Weinberg equilibrium (HWE) expectation in control subjects was assessed by a chi-square (*χ*^2^) test implemented in PyPop for each locus 8. The association between each genetic variant and disease risk was estimated by the odds ratio (OR) and 95% confidence interval (CI) per allele, assuming a log-additive genetic model using unconditional logistic regression adjusting for age at recruitment and study design (CIS vs. invasive carcinoma) in 961 cervical cancer patients and 1725 controls.

For genetic variants that showed evidence of association, the heterozygous and homozygous carriers of the variant allele were compared with the noncarriers, respectively. Analyses were also conducted after stratifying for study design or tumor stage (CIS vs. invasive carcinoma) and age at recruitment (age<36 and age≥36), adjusting for age at recruitment and study design (CIS vs. invasive carcinoma) when necessary. Heterogeneity of odds ratios across the stratification groups was assessed using the Cochran Q test. The potential interaction between each genetic variant and study design (CIS vs. invasive carcinoma) was evaluated with the interaction model by additionally including interaction term between genotypes and study design. Statistical analyses were all performed using SAS 9.3 software (SAS Institute, Cary, NC), with two-sided tests. A Bonferroni correction for multiple tests was applied and gave a *P* value of 7.1 × 10^−3^ as the cutoff for statistical significance based on seven independent genetic variants tested (SNP rs2516448 is in perfect LD with A5.1 of *MICA*, hence they represent one independent test).

## Results

The characteristics of the cervical cancer patients and cancer-free controls enrolled in the study are described in Table 1. Overall, there was no significant difference in age between the cervical cancer patients and the control subject (*P* = 0.22), suggesting that matching based on age was adequate. The median age was 36 years for both cases and controls. Of the 961 cervical cancer patients, 827 (86.06%) had a diagnosis of CIS and 134 (13.94%) of invasive carcinoma.

Table 2 summarizes the estimates of the main effects for each SNP. Genotype frequency distributions in the control subjects were consistent with those expected from the HWE model for all SNPs (all *P* > 0.05). The variant allele T of rs2516448 in the MHC class I region was significantly associated with increased risk of cervical cancer (OR = 1.33, 95% CI = 1.19–1.49; *P* = 5.8 × 10^−7^), whereas the variant allele C of rs9272143 in the MHC class II region was strongly associated with decreased risk of cervical cancer (OR = 0.73, 95% CI = 0.65–0.82; *P* = 1.6×10^−7^). Both the direction and magnitude of these associations were in accordance with our previous findings 5. In contrast, there was no association between rs3117027 and risk of cervical cancer (OR = 0.99, 95% CI = 0.88–1.12 for variant allele A; *P* = 0.86). The LD between these three SNPs was very weak (*r*^2^ = 0) (Supplementary Table S2), consistent with previous study 5.

**Table 2:** Summary estimates of the main effects of the selected variants at 6p21.3 reported to independently associate with cervical cancer

Loci	SNP	Position^1^	Nearby gene	Alleles^2^	HWE^3^	Genotyping Rate (%)	Cases		Controls		Association 6	
							*N*^4^	Frequency^6^	*N*^4^	Frequency^6^	OR (95% CI)	*P*
Locus 1	rs9272143	32600803 (class ΙΙ)	*HLA-DRB1, HLA-DQA1*	T > C	0.54	99.96	960	0.38	1725	0.45	0.73 (0.65–0.82)	1.6 × 10^−7^
Locus 2	rs2516448	31390410 (class Ι)	*MICA*	C > T	0.14	99.55	955	0.60	1719	0.52	1.33 (1.19–1.49)	5.8 × 10^−7^
Locus 3	rs3117027	33089623 (class ΙΙ)	*HLA-DPB2*	C > A	0.29	99.63	960	0.30	1716	0.31	0.99 (0.88–1.12)	0.86

HWE, Hardy–Weinberg equilibrium; MAF, minor allele frequency; OR, odds ratio; CI, confidence interval.

1 Genome build 37.3, (GRCh37/hg19) Assembly.

2 Wild-type allele > Variant allele.

3 *P* values for Hardy–Weinberg equilibrium in the controls.

4 Number of samples that were successfully genotyped for specified SNP in cervical cancer patients and control subjects, respectively.

5 Frequency of the variant alleles in the cases and controls, respectively.

6 Odds ratios and 95% confidence intervals for the variant allele in log-additive model were derived from unconditional logistic regression adjusting for age at recruitment and study design (carcinoma in situ vs. invasive carcinoma). Two-sided *P* values correspond to the odds ratios.

The allele frequencies of the *MICA* exon 5 microsatellite in cervical cancer patients and control subjects are shown in Table 3. Analysis showed that *MICA-A5.1* (G insertion of rs67841474) had the highest frequency in both patients (60%) and control subjects (52%). In accordance with our previous finding 5, this microsatellite allele was in perfect LD (*D'* = 1, *r*^2^ = 1) with the risk allele T of rs2516448 in both cases and controls, and showed a comparable association with susceptibility to cervical cancer (OR = 1.34, 95% CI = 1.20–1.50; *P* = 3.8 × 10^−7^) as the rs2516448 T allele. In the overlapping 943 cervical cancer patients and 1683 cancer-free control subjects, the OR (95% CI) was 1.34 (1.20–1.51) for both A5.1 and the T allele of rs2516448. In addition, significant protective effects were seen for *MICA-A4* (OR=0.80, 95% CI = 0.68–0.94; *P* = 6.7×10^−3^) and *MICA-A5* (OR = 0.60, 95% CI = 0.50–0.72; *P* = 3.0 × 10^−8^). No correlation was found between rs9272143 and rs3117027 and alleles of *MICA* exon 5 microsatellite (*r*^2^ = 0) (Supplementary Table S2).

**Table 3:** Association between *MICA* microsatellite and cervical cancer

Alleles	Allele count (allele frequency)		Association^2^	
	Cases (Total alleles =1898)^1^	Controls (Total alleles =3378)^1^	OR (95% CI)	*P*
A4	261 (0.14)	566 (0.17)	0.80 (0.68–0.94)	6.7 × 10^−3^
A5	179 (0.09)	500 (0.15)	0.60 (0.50–0.72)	3.0 × 10^−8^
A5.1	1138 (0.60)	1771 (0.52)	1.34 (1.20–1.50)	3.8 × 10^−7^
A6	182 (0.10)	290 (0.09)	1.12 (0.92–1.36)	0.25
A9	138 (0.07)	251 (0.07)	0.98 (0.79–1.22)	0.87

OR, odds ratio; CI, confidence interval.

1 Total allele counts in cervical cancer patients and control subjects, respectively.

2 Odds ratios and 95% confidence intervals for each allele in log-additive model were derived from unconditional logistic regression adjusting for age at recruitment and study design (carcinoma in situ vs. invasive carcinoma). Two-sided *P* values correspond to the odds ratios.

**Figure 1:**
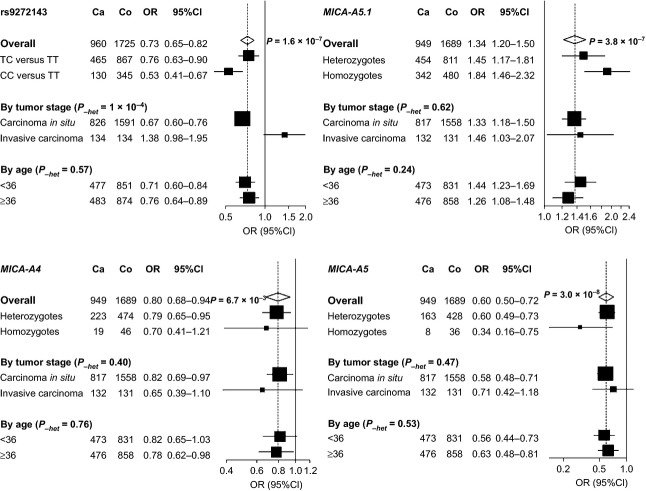
Stratified analysis of association between rs9272143, *MICA-A5.1, -A4,* and *-A5* and risk of cervical cancer. Unless specified, the odds ratios (ORs) and 95% confidence intervals (CIs) of per variant allele (log-additive model) and per genotype were calculated using unconditional logistic regression with adjustment of age at recruitment and study design (carcinoma in situ vs. invasive carcinoma) when appropriate. *P* for heterogeneity (*P*_*-het*_) was derived from the Cochran Q test. Squares represent odds ratios; size of the square represents inverse of the variance of the log odds ratio; horizontal lines represent 95% confidence intervals; diamonds represent overall estimate; solid vertical lines represent an odds ratio of 1; dashed vertical lines represent the overall odds ratios. Ca, case subject; Co, control subject.

An allelic dosage effect on cervical cancer risk was observed for variant at rs9272143 in the MHC class II region and the *MICA*-*A4*, *-A5* and *-A5.1* alleles, when comparing the heterozygous and homozygous carriers of the variant allele with the noncarriers (Fig. 1). In particular, *MICA-A5*.1 homozygotes had a 1.84-fold increased risk of developing cervical cancer (OR = 1.84, 95% CI = 1.46–2.32; *P* = 3.2 × 10^−7^), whereas *MICA-A5* homozygotes had nearly threefold protection against cervical cancer (OR = 0.34, 95% CI = 0.16–0.75; *P* = 7.0×10^−3^) as compared to noncarriers, respectively. No statistically significant heterogeneity was observed by age for any of the genetic variants. However, the variant of rs9272143 in the MHC class II region showed strong heterogeneity when stratifying by study design (or tumor stage) (*P*_*-het*_ = 1 × 10^−4^), with little evidence for association in invasive carcinoma (OR = 1.38, 95% CI = 0.98–1.95; *P* = 0.07), although the number of subjects with invasive cancer was limited. By contrast, there was little evidence for heterogeneity by tumor stage for the *MICA* variants, and a statistically significant association with *MICA-A5.1* was observed in invasive carcinoma (OR = 1.46, 95% CI = 1.03–2.07; *P* = 0.03). Consistently, statistically significant interaction was observed between tumor stage and rs9272143 (*P* = 4.4 × 10^−4^), but not between tumor stage and *MICA* alleles (all *P* > 0.05).

## Discussion

We replicated the associations of cervical cancer with rs9272143 located in the MHC class II region as well as with rs2516448 and *MICA-A5.1* in the class I region identified in our previous GWAS, with ORs of similar magnitude to that previously reported 5. We also identified protective effects of both the *MICA-A4* and *MICA-A5* alleles against cervical cancer. None of these variants showed heterogeneity by age of onset. The association between *HLA-DPB2* variant rs3117027 and risk of cervical cancer was not replicated in this study.

SNP rs9272143 is located 4.38 kb upstream of *HLA-DQA1* and 43.19 kb upstream of *HLA-DRB1*. It has recently been identified as a *cis*-expression quantitative trait locus (*cis*-eQTL), with the T allele being associated with decreased expression of *HLA-DRB1* as compared to the C allele 9. *HLA-DRB1* belongs to the HLA class II *β*-chain paralogs, which encodes the *β*-chain of the peptide-antigen receptor HLA-DR. It is expressed in Langerhans cells (LC), the antigen presenting cells of squamous epithelia in the cervix, and plays a central role in the cell-mediated immune response by presenting processed foreign antigens to CD4+ helper T-lymphocytes 10. CD4+ T-cell activation results in the secretion of a variety of small proteins, or cytokines. Our study points to the importance of expression level of *HLA-DRB1* in cervical carcinoma. Impaired class II gene expression 11–13 and a reduced number of LC have been reported in genital HPV infections 14,15 and in lesions due to HPV 16. The increased incidence and progression of HPV infections in immunosuppressed individuals illustrates the critical importance of the CD4+ T-cell-regulated cell-mediated immune response in the resolution and control of HPV infection 10,17. Regression of anogenital warts is accompanied histologically by a CD4+ T-cell-dominated Th1 response. Failure to develop effective cell-mediated immune response to clear or control infection results in a persistent infection and, in the case of the oncogenic HPVs, an increased probability of progression to CIS and invasive carcinoma 17. Therefore, *HLA-DRB1* may be involved in the key event of establishing a robust defense against HPV infection through antigen presentation to CD4+ helper T-lymphocytes. It is, hence, biologically plausible that carriers of the C allele of rs9272143, which have higher expression level of *HLA-DRB1* are less susceptible to cervical cancer. However, it is unknown whether rs9272143 is the pathogenic variant or other functional variant(s) which affect(s) the expression of *HLA-DRB1* could be responsible for this signal. Functional studies would help to provide insight into it.

*MICA* encodes a membrane-bound protein, which acts as a ligand to stimulate an activating receptor, NKG2D, expressed on the surface of essentially all human natural killer (NK), *γδ* T, and CD8^+^ *αβ* T cells 18–20. Normally, MICA is constitutively expressed in low levels on epithelial cells in the gut and thymus, endothelial cells, fibroblasts, and monocytes 21–23. But it is upregulated or expressed de novo in stressed conditions, such as during viral and bacterial infections 20,24,25, heat shock 22, DNA damage response 26, oncogenic transformation 18,19, and in autoimmune conditions 27. MICA serves as signal of cellular stress, and engagement of NKG2D by MICA triggers NK cells, and costimulates some *γδ* T cells and antigen-specific CD8^+^ *αβ* T cells, resulting in a range of immune effector functions, such as cytotoxicity and cytokine production 21,28. The recognition of the MICA molecule by the NKG2D receptor enables immune cells to identify and attack infected or transformed cells without the need of MHC class I expression or antigen recognition 29. Thus, the MICA/NKG2D interaction is an effective mechanism for immunosurveillance. This strong selection pressure seems to have led tumor cells to evolve mechanisms to minimize or avoid the response mediated by NKG2D by shedding MICA from the cell surface 30,31. The shedding of MICA has been reported to be mediated by metalloproteinases through proteolytic cleavage of the extracellular parts and palmitoylation of two cysteine residues in the cytoplasmic tail of MICA was found to be necessary for efficient cleavage 31,32. The shedding of soluble MICA by human tumors not only hinders recognition of the MICA-expression tumor cells, but also results in systemic downregulation of NKG2D on NK and CD8^+^ T cells, and evasion of NKG2D-mediated immune recognition 30,31.

The SNP rs2516448 is located 7.32 kb downstream of the *MICA* gene and its effect is independent of previously known associations with HLA alleles/haplotypes 5. The T allele of rs2516448 that increased the susceptibility to cervical cancer is in perfect LD with the *MICA-A5.1* allele, which encodes a truncated protein lacking part of the TMD and the whole cytoplasmic tail and is most commonly seen in the MICA*008 allele 6. Suemizu et al. 33 found that the cytoplasmic tail-deleted *MICA-A5.1* gene product was aberrantly transported to the apical surface of human intestinal epithelial cells instead of the basolateral surface where the interaction with intraepithelial T and NK lymphocytes takes place. Thus, *MICA-A5.1* carriers may have an aberrant immunological surveillance by NK and T cells. Meanwhile, in contrast to other MICA alleles that are shed as truncated soluble species after proteolysis by metalloproteinases, the protein translated from the *MICA-A5.1* allele is released from cells as a membrane-anchored full-length molecule in exosomes due to the lack of the two cysteines required for proteolytic shedding. Incubation of NK cells with the *MICA-A5.1* (MICA*008) containing supernatant triggers significantly more NKG2D downregulation than the MICA*019 culture supernatant. Strikingly, incubation with exosomes containing *MICA-A5.1* (MICA*008) also impairs NK-cell cytotoxicity 34. Taken together, the preceding evidence might explain the result in this study that individuals carrying the *MICA-A5.1* allele have a predisposition for their infected or transformed cells to escape from attack by immune cells. The *MICA-A5.1* allele has also been associated with different autoimmune diseases and other tumor forms 35–42, supporting its role in immune response and tumor development.

In contrast to *MICA-A5.1*, alleles encoding only four (*MICA-A4*) or five (*MICA-A5*) Ala residues in the TMD of MICA protein, respectively, were found to confer protection against cervical cancer. In particular, a very strong protective effect was observed for the A5/A5 genotype. Further studies with larger sample sizes are warranted to verify this result, given the limited number of *MICA-A5* homozygotes in this study. Interestingly, in breast cancer, *MICA-A4* and *MICA-A5* have also shown a protective effect 41,42. The mechanism by which *MICA-A4* and *MICA-A5* protect against cervical cancer is yet unknown. The nearby HLA-B*0702 has been reported to be associated with cervical cancer risk 5,43,44. However, in a recent study, we found that the association with HLA-B*0702 was actually driven by the joint effects of both rs9272143 and *MICA-A5.1* (unpublished data), suggesting that HLA-B*0702 is unlikely to be the causal variant responsible for the association with *MICA-A4* or *MICA-A5*. These short tandem repeats are not located in any of the extracellular domains and do not directly affect the putative binding site of MICA with NKG2D. As the amino acids encoded by the microsatellite are situated in the TMD of the molecule, it is possible that certain alleles provide a more stable anchoring of MICA to the cell surface and therefore permit better binding to NKG2D and, as a consequence, a more efficient NKG2D-mediated immunosurveillance 41,45. It is also worth noting that *MICA-A4* is in high LD with the amino acid substitution of Glycine (Gly) by Tryptophan (Trp) at position 14 in the *α*1 domain and *MICA-A5* is in high LD with the amino acid substitution of Gly by Serine (Ser) at position 175 in the *α*2 domain of MICA in the IMGT/HLA database 46. The top surface of the MICA *α*1-*α*2 platform has been found to interact directly with NKG2D 47,48. Further studies are warranted to determine whether these amino acid changes could affect the binding affinity of MICA to the NKG2D receptor and whether they are responsible for the associations with *MICA-A4* and *MICA-A5*.

The SNP rs3117017 resides in the pseudogene *HLA-DPB2,* but close to the functional gene *HLA-DPB1*, which encodes the *β*-chain of the peptide-antigen receptor HLA-DP. In contrast to our previous GWAS of cervical cancer, we did not observe a significant association between this SNP and risk of cervical cancer. It is noteworthy that the effect size of rs3117027 variant was much smaller (OR = 1.25) than the other two hits and the *P* value was close to genome-wide significance threshold in the initial GWAS discovery cohort (*P* = 3.1 × 10^−6^). It is possible that this study lacks sufficient statistical power to detect its modest effect on susceptibility for cervical cancer. Further studies with larger sample sizes are needed to draw a firm conclusion. On the other hand, the subjects enrolled in this study are from northern Sweden only, while the subjects in the GWAS were a national collection. The lack of replication may indicate no direct association between rs3117027 and cervical cancer. One cannot dismiss the possibility, however, that the LD between rs3117027 and the putative causal variant varies between ethnically distinct populations.

This study has several limitations. First, the number of invasive cervical cancer cases (134) included in the study is modest. Although we have observed evidence for heterogeneity by tumor stage for rs9272143, the statistical power is insufficient to draw any firm conclusion. Future studies with larger numbers of invasive cancers are warranted to validate this finding. Second, except for age, information on other risk factors for cervical cancer such as HPV infection, parity, oral contraceptive use, and tobacco smoking 49, which might modify the effects of the susceptibility loci, was not available in our study. Possible interactions between the susceptibility loci and these risk factors should be thoroughly investigated in future studies.

In summary, associations identified in our previous GWAS with rs9272143 in the MHC class II region, as well as rs2516448 and the *MICA-A5.1* allele in the class I region were replicated in a northern Swedish population, providing credible evidence that these genetic variants influence susceptibility to cervical cancer. We also identified a reduction in risk associated with the *MICA-A4* and *MICA-A5* alleles. Our results do not support previous suggestion that *HLA-DPB2* variant rs3117027 is positively associated with cervical cancer. The association with rs2516448 seems to be driven by the functional allele *MICA-A5.1*. However, it is unknown whether other allele combinations not measured by this study could be responsible for the signals of rs9272143 as well as *MICA-A4* and *MICA-A5* alleles. Further functional studies are warranted to identify the causal variant (s) responsible for these signals and their functional effect (s).

## Supporting Information

**Table S1.** Genotyping information for SNPs and MICA microsatellite. **Table S2.** Linkage disequilibrium (D′ and r^2^) between SNPs and alleles of MICA microsatellite in controls
