# Supplementary material for: A variant upstream of HLA-DRB1 and multiple variants in MICA influence susceptibility to cervical cancer in a Swedish population
Source: Cancer Med. 2014 Jan 7;3(1):190–8. doi: 10.1002/cam4.183 (PMC3930404; doi:10.1002/cam4.183)
Supplement: Supplementary file 1 — Table S1. Genotyping information for SNPs and MICA microsatellite. Table S2. Linkage disequilibrium (D′ and r2) between SNPs and alleles of MICA microsatellite in controls [file cam40003-0190-sd1.docx]

**Supplementary Table S1. Genotyping information for SNPs and *MICA* microsatellite**

| **SNP** | **Assay** | **Primer sequences (5’-3’)** | **Probes/Extension primers** |
| --- | --- | --- | --- |
| rs2516448 | Taqman | Forward:TCCCTTTTTGCATCCCTCTACTAGA | VIC: CTGCTTCAGCTATATGTG |
|  |  | Reverse: CACACACATACACTCACATATACACACT | FAM: TGCTTCAGCTGTATGTG |
| rs9272143 | FP-TDI ^a^ | Forward: TGCCAAACCTATTGATGCTACA | Extension primer1:GCTGAAAAACACAATTTTTTTGAAG |
|  |  | Reverse: AGCCACAGAAGCACTAAGACATT | Extension primer2:GCTGAAAAACAAAATTTTTTTGAAG |
| rs3117027 | FP-TDI ^a^ | Forward: GCGGTTAGAGTTTGGTTTAGGA | Extension primer1:TGAAACACCTGAGTTAAATTCTGGA |
|  |  | Reverse: GCCCACTGTTCTTTCTCTATACTTT | Extension primer2:TGAAATACCTGAGTTAAATTCTGGA |
| Microsatellite | PCR ^b^ | Forward: CCTTACCATCTCCAGAAACTGC | None |
|  |  | Reverse: CCTTTTTTTAGGGAAAGTGC |  |

^a^ FP-TDI, template-directed dye-terminator incorporation with fluorescence-polarization detection (FP-TDI).

^b^ PCR, the polymerase chain reaction.

**Supplementary Table S2. Linkage disequilibrium (*D´* and *r^2^*) between SNPs and alleles of *MICA* microsatellite in controls**

| **Variant** | **rs2516448** | **rs9272143** | **rs3117027** | **A4** | **A5** | **A5.1** | **A6** | **A9** |
| --- | --- | --- | --- | --- | --- | --- | --- | --- |
| **rs2516448** | — | 0.06 | 0.01 | 1.0 | 1.0 | 1.0 | 1.0 | 1.0 |
| **rs9272143** | 0 | — | 0.11 | 0.17 | 0.16 | 0.06 | 0.07 | 0.23 |
| **rs3117027** | 0 | 0 | — | 0.03 | 0.03 | 0.01 | 0.10 | 0.28 |
| **A4** | 0.22 | 0 | 0 | — | 0 | 0 | 0 | 0 |
| **A5** | 0.19 | 0 | 0 | 0 | — | 0 | 0 | 0 |
| **A5.1** | 1.0 | 0 | 0 | 0 | 0 | — | 0 | 0 |
| **A6** | 0.10 | 0 | 0 | 0 | 0 | 0 | — | 0 |
| **A9** | 0.09 | 0 | 0 | 0 | 0 | 0 | 0 | — |

*D*’ values are given above the diagonal; *r^2^* values are given below the diagonal.
